# Deep learning for tumor margin identification in electromagnetic imaging

**DOI:** 10.1038/s41598-023-42625-w

**Published:** 2023-09-23

**Authors:** Amir Mirbeik, Negar Ebadi

**Affiliations:** 1 RadioSight LLC, Hoboken, NJ 07030 USA; 2 Department of Electrical and Computer Engineering, Stevens Institute of Technology, 1 Castle Point Ter, Hoboken, NJ 07030 USA; 3 Stanford University School of Medicine, Stanford, CA USA

**Keywords:** Preclinical research, Translational research, Hardware and infrastructure, Image processing, Machine learning

## Abstract

In this work, a novel method for tumor margin identification in electromagnetic imaging is proposed to optimize the tumor removal surgery. This capability will enable the visualization of the border of the cancerous tissue for the surgeon prior or during the excision surgery. To this end, the border between the normal and tumor parts needs to be identified. Therefore, the images need to be segmented into tumor and normal areas. We propose a deep learning technique which divides the electromagnetic images into two regions: tumor and normal, with high accuracy. We formulate deep learning from a perspective relevant to electromagnetic image reconstruction. A recurrent auto-encoder network architecture (termed here DeepTMI) is presented. The effectiveness of the algorithm is demonstrated by segmenting the reconstructed images of an experimental tissue-mimicking phantom. The structure similarity measure (SSIM) and mean-square-error (MSE) average of normalized reconstructed results by the DeepTMI method are about 0.94 and 0.04 respectively, while that average obtained from the conventional backpropagation (BP) method can hardly overcome 0.35 and 0.41 respectively.

## Introduction

Surgical resection is the mainstream treatment for solid tumors (tissues that normally do not contain cysts or liquid areas). The resected specimens are sent to pathology for evaluation and subsequently an additional piece of tissue is removed if cancer is observed in the margins of the resected pieces. In this procedure, sampling error may be present as less than 2% of the total sample is normally examined microscopically due to tissue processing and sectioning^1^. Therefore, the presence of tumor at the margins is sometimes underestimated or even missed. On the other hand, often times some normal tissue is unnecessarily removed during surgery. Since lesions generally occur on sun-exposed body parts, for instance the face, minimizing tissue loss is important for improving the quality of life of cancer patients.

However, the current methods to distinguish malignant from healthy tissue are primarily limited to tactile and visual cues as well as the surgeon's experience. As a result, a positive surgical margin or the presence of residual tumor may be left behind after resection. It is well-documented that the presence of residual tumor can negatively impact treatment outcomes and survival, as well as pose an economic burden^2^.

Imaging techniques would be highly valuable in assisting surgeons to identify tumor margins pre- or intra-operatively to evaluate the remainder of the resected lesion on the body. Several non-invasive imaging tools such as neuro-navigation, magnetic resonance imaging (MRI), ultrasound (US), Raman spectroscopy (RS), and optical fluorescence imaging (FLI) have been developed for this purpose^3–14^. However, they each have limitations on their uses and provide optimal outcomes only under certain conditions. No imaging tool has been widely adopted in the clinics. There is a definite need for an imaging tool that could visualize tumor profiles with sufficient contrasts throughout the depth of the skin at an affordable cost.

In Ref.^15^, the authors developed a high-resolution millimeter-wave imaging (HR-MMWI) system for early-stage detection of skin cancer. In-vivo measurements of more than 136 cancerous and benign skin lesions were performed. In particular, the diagnosis of skin lesions with clinical interest, i.e., cancer/precancer versus benign lesions, was concerned. The findings established that real-time millimeter-wave imaging can distinguish between malignant tissues and benign skin lesions with high diagnostic accuracy (97% sensitivity and 98% specificity). As the next step, the capability of the system in identifying tumor margins pre- or intra-operatively needs to be verified. This capability will potentially simplify the tumor removal surgery to a single-layer excision procedure by visualizing the borders of the cancerous tissue for the surgeon. Nonlinear electromagnetic (EM) inverse scattering is the conventional imaging technique for accurately reconstructing deictic properties of a target. However, there are important challenges arising from intrinsic strong nonlinearity, ill-posed-ness, and high computational costs.

To tackle these difficulties, in this work, we propose a novel method for tumor margin identification in 3-D electromagnetic imaging. To identify tumor margins, the border between the normal and tumor parts needs to be identified. Therefore, the images need to be segmented into tumor and normal areas. The proposed method is a learned technique which divides the electromagnetic images into two regions, i.e. tumor and normal, with high accuracy. We establish a fundamental connection between a deep neural network architecture and iterative methods utilized for nonlinear EM inverse scattering problems. Inspired by this connection, we then develop a novel DNN architecture tailored for tumor margin identification (termed here DeepTMI), which consists of a cascade of multilayer neural network modules.

The performance of the proposed method is validated by an experimental demonstration. We examine the effectiveness of the algorithm by segmenting the reconstructed images of an experimental skin phantom^16^. Specifically, it is shown that the method is a promising tool for efficiently tackling nonlinear inverse scattering problems, which are impractical to be solved by using conventional methods.

## Problem statement

Over the past few years, deep learning has consolidated as one of the most powerful approaches in several areas of regression and classification, due to ease of availability of vast amounts of data and the ever-increasing computational power^17,18^. Deep neural networks (DNN) specifically have attracted attention in image processing and computer vision, such as semantic segmentation^19^ depth estimation^20^, image deblurring^21^, and image super resolution^22,23^. DNN was also demonstrated to be advantageous over traditional machine learning approaches in the automated analysis of high-content microscopy data^24^. Deep learning was also shown to aid the design and realization of advanced functional materials^25^ and high-accuracy reconstruction from compressed measurements^26,27^. Most recently, DNN algorithms have been applied in biomedical imaging, e.g., magnetic resonance imaging^28^, X-ray computed tomography^29^, and computational optical imaging^30,31^. It has been empirically found that NN-based^32,33^ and DNN-based strategies can outperform conventional image reconstruction techniques in terms of improved image quality and reduced computational costs^28–33^.

Motivated by the capability of neural networks, this paper focuses on adapting a new architecture, namely DeepTMI to iterative optimizations utilized for the nonlinear EM inverse scattering problems. The proposed method is a learned technique which divides the electromagnetic images into two regions, i.e. tumor and normal, with high accuracy and approximately characterizes the multi-scattering physical mechanism. The proposed neural network module is a straightforward extension of the conventional neural networks^22^, which is an end-to-end map from an input rough image to the refined solution of a nonlinear inverse scattering problem. The input data of the first module of the network comes from the backpropagation (BP) image. For the remaining modules of the network, the input is the output of the previous module.

We initiate the discussion by unveiling the connection between the DNN architecture of interest and iterative methods for nonlinear EM inverse scattering. Since the iterative solution of a nonlinear EM inverse scattering requires convolutions and should account for nonlinearities, DNN may offer an efficient alternative solution.

### Connection between DNN and nonlinear EM inverse scattering

**Figure 1:**
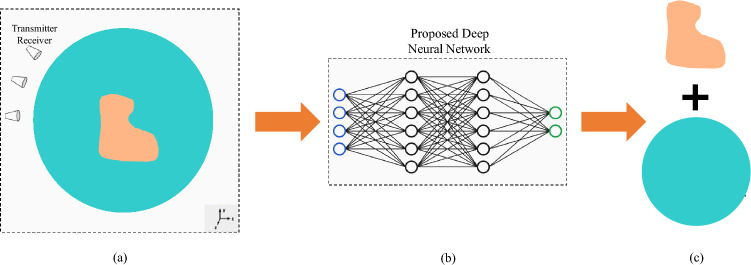
Basic configuration of the developed deep learning-based solver. (**a**) The complex-valued input comes from conventional reconstruction algorithms, and the output is the image of inverse scattering. The proposed method (**b**) divides the electromagnetic image into two regions, tumor and normal, with high accuracy as shown in (**c**).

With reference to the measurement configuration in Fig. 1a, we illustrate our strategy in the context of a multiple-input multiple-output measurement configuration. For computational imaging, the investigation domain is uniformly divided into pixels such that the total electric fields, the contrast currents, and the contrast functions are assumed uniform in each pixel. In the area of image processing, it has become a consensus that most of the natural images have some structure. This underlying structure allows for a sparse representation in some transformed domain, which also assists in regularization.

In ultra-wideband imaging also, the presence and location of significant backscatters such as a malignant tumor is identified through solving the inverse problem of (1), i.e. a reconstruction technique. The main challenge in image reconstruction is devising an algorithm which provides high resolutions and suppressions of strong artifacts and noise. Various image reconstruction algorithms have been proposed for ultra-wide band imaging^34,35^. These techniques essentially employ the delay-and-sum (DAS) concept (with various modifications) in which the backscattered signals are time-shifted and summed to create a synthetic focal point of the target.

We can model the received signal ($$S)$$ as follows^16^:

1 $${\text{S}} = [S({\text{r}})] = {\mathcal{F}}\left[ s \right] = \iint s({\text{x}},f)\exp \left\{ { - 2jkR^{{\text{x}}} ({\text{r}})} \right\}dfd{\text{x}} = {\text{Fs}}$$

where x is the three-dimensional position of the scatterer, *f* is the frequency, $s(\mathrm{x},f)$ is the reflectivity function of scatterer, ${R}^{x}$ is the electrical distance between the scatterer and the scanning point r, and *k* = *ω*/*c* is the wavenumber (*ω* is the temporal angular frequency and *c* is the speed of light).

Iterative sparsity promoting algorithms have been recently proposed for image reconstruction. As demonstrated in (1), the scattering data of a target at high frequencies can be represented as a sum of responses from individual scattering centers. These scatterers are sparsely distributed in the sensing scene i.e., most of the coefficients in the target’s signature vector are zeros or nearly zeros. We use an iterative approach to reconstruct the level set and corresponding reflectivity profile from a set of measured and computed data. A common non-linear image formation method is to regularize a quadratic data likelihood function and solve the following optimization problem.

The use of sparsity in signal processing frequently calls for the solution to the minimization problem^36^:

2 $${\hat{\text{s}}} = {\text{Argmin}} C({\text{s}}) = \frac{1}{2}\left\| {{\text{Fs}} - {\text{S}}} \right\|_{2}^{2} + \lambda \left\| {\text{s}} \right\|_{1}$$

where *C* is a regularized least-squares cost function used to assess the similarity of the measured received signal (S) to the total computed reflected signal for the reconstructed reflectivity profile. The problem in (2) can be solved iteratively using a forward–backward splitting algorithm. First a gradient descent update is performed for the smooth component of the objective function (the $\mathcal{l}$^2^-norm term) and a projection is done for the non-smooth term. This results in the following iteration:

3 $${\text{s}}^{k + 1} = {\mathcal{P}}_{\lambda } \left( {{\text{s}}^{{\text{k}}} - \alpha {\text{F}}^{{\text{H}}} \left( {{\text{Fs}}^{{\text{k}}} - {\text{S}}} \right)} \right)$$

where ${\mathcal{P}}_{\lambda }$ is the shrinkage operator and performs a soft thresholding of the input (i.e., the proximity operator for the $\mathcalligra{l}$^1^-norm).

### DNN for nonlinear EM inverse scattering

The iterative imaging algorithm of (3) emulates a feed-forward neural network which is the most general architecture for deep learning. In a feed-forward neural network, each layer of the network represents an iteration and the inner product with a weight matrix plus bias is fed as the input. The neural network is formed by unfolding the iterative optimization method^37^. Motivated by this recent application of deep learning to inverse problems and the power of deep neural networks to approximate non-linear mappings, we investigate the use of deep learning in reconstructing the level-set function. A level set is a real-valued function defined in the whole imaging domain. An example of a single level set segmenting a domain into distinct regions is shown in Fig. 1a, where The goal is to accurately identify the sections with dielectric properties corresponding to cancer tissue, and regions with dielectric properties corresponding to normal tissue as shown Fig. 1b,c.

After demonstrating the natural connection between the DNN architecture and nonlinear EM inverse scattering, we now develop a complex valued DNN to solve the nonlinear EM inverse scattering problem. For the sake of DNN computational complexity, the proposed DeepTMI can be designed as a cascade of CNN modules where the input data of the network comes from the BP image. For the remaining modules of the network, the output of the previous CNN module is the input of the next module. Each module consists of several up-sampling convolution layers, each layer consisting of three steps: in the first step, the input is convolved with a set of learned fitters, resulting in a set of feature (or kernel) maps; in the second step, the maps undergo a point-wise nonlinear function, resulting in a sparse outcome; an optional third down-sampling step (termed as pooling) is applied on the result to reduce its dimensions, thus forming the multilayer structure.

### Algorithm implementation

Deep learning has recently become immensely popular in a wide variety of traditional signal processing tasks such as image segmentation^38^, denoising^39^, and point source localization in the presence of noise^40^, displaying extremely impressive results. In the area of remote sensing and RF imaging, deep learning is mostly applied to terrain surface classification^41,42^, target segmentation^43,44^, object detection^45^, antenna selection in cognitive radar^46^, interference mitigation^47^, vehicle detection^48^ in automotive applications, and activity recognition in indoor monitoring^49–51^. In all these works, the neural networks operate on images that have already been formed using other image formation approaches. Despite the success in optical imaging, the huge potential of deep learning in UWB image reconstruction remains locked.

Here, we review the most relevant deep learning models, point out possible pitfalls by analyzing special characteristics of electromagnetic scattering data, evaluate the performance of the state-of-the-art of deep learning when applied to remote sensing and inverse problems, and conclude with an optimized framework to be used in our imaging experiments.

#### CNNs

Convolutional neural networks (CNNs) have attracted worldwide attention and are currently used for many image understanding tasks, such as image classification, object detection, and semantic segmentation. Residual neural network (ResNet)^52^, U-Net^53^, and DenseNet^54^ were the subsequent major CNN architectures. Their main feature concerned the idea of connecting not only neighboring layers but any two layers in the network by using skip connections. This helped reduce information loss across networks, mitigated the problem of vanishing gradients, and facilitated the design of deeper networks. U-Net is among the most common image segmentation networks.

#### RNNs

Besides CNNs, recurrent neural networks (RNNs)^55^ are a major class of deep networks. Their main building blocks are recurrent units, which take the current input and the output of the previous state as their input. They provide state-of-the-art results for processing data of variable lengths, including text and time-series information. Their weights can be replaced with convolutional kernels for visual processing tasks, such as image captioning and predicting future frames/points in visual time-series data.

#### GANs

Proposed by Ian Goodfellow et al.^56^, generative adversarial networks (GANs) are among the most popular inventions in the field of deep learning. Based on game theoretic principles, they consist of two networks, i.e. a generator and a discriminator. The generator’s objective is to learn a latent space through which it can create samples from the same distribution as the training data, while the discriminator tries to learn to distinguish whether a sample is from the generator or the training data.

The learning procedure in all the above-mentioned networks takes place by optimizing a cost functional (typically an *l*^2^-norm of the mismatch between the network output and the ground truth) with respect to network weights. This optimization is mostly carried out using the stochastic gradient descent (SGD) algorithm. The optimization problem is typically high-dimensional and highly non-convex, often consisting of many saddle points and non-optimal local minima. Therefore, a critical aspect would be the initialization of the network weights. Prior knowledge of the input-to-output mapping initializing the back propagation increases the chance of reaching an optimal solution. In this research, we will use the physical forward model of UWB imaging to obtain suitable initialization*.*

The weights of the network provide a parametrization of the operation that the network performs, whereas the non-linear units introduce the capacity to approximate complex, non-linear mappings between input and output spaces. These sophisticated transformations are generally explained in terms of the universal approximation theorem or probabilistic inference^37,57^. Although the universal approximation theorem states that a single hidden layer neural network is a universal approximator, the depth of the architecture introduces new strengths and capabilities^58^. This results in deep networks gaining more expressive power and the ability to represent more complex mappings. This is a direct consequence of the fact that ANNs are formed by the composition of multiple non-linear mappings.

The output at each layer of the neural network can be characterized as

4 $${\text{h}}^{(k + 1)} = \sigma (\tilde{W}^{k} \cdot \tilde{h}^{{\text{k}}} + {\text{w}}_{{\text{b}}}^{{\text{k}}} )$$

where $\widetilde{\text{h}}$^*k*^ is the input vector, $\widetilde{W}$^*k*^ is the weight matrix, and ${\mathrm{w}}_{\mathrm{b}}^{k}$ is the bias weight vector of the *k*th layer, $\sigma$ is an element-wise, non-linear activation function, and h^*k*+1^ is the output of the *k*th layer, and subsequently the input of the (*k* + 1)th layer of the network.

In our case, we use the auto-encoder framework combined with a recurrent neural network (RNN) to form a recurrent auto-encoder^59^. An RNN fundamentally differs from other architectures by fixing the weight matrix across all layers. Essentially, an RNN can be interpreted as an implementation of an iterative algorithm for a fixed number of iterations. This makes it ideal as an architecture for applying deep learning to the level-set image reconstruction problem as it allows for the utilization of existing image reconstruction methods such as the DAS algorithm. Furthermore, RNNs can be trained in a similar fashion to other network architectures. Specifically, a stochastic gradient descent with the DAS algorithm can be used to analytically calculate the derivatives.

We set up the RNN by unfolding the algorithm up to a fixed number of iterations. To demonstrate how (3) relates to (4), we first define **K** = **I**–*α***F**^*H*^**F**. Using this notation, we can express (3) as:

5 $${\text{s}}^{k + 1} = {\mathcal{P}}_{\lambda } \left( {{\text{Ks}}^{{\text{k}}} + \alpha {\text{F}}^{{\text{H}}} {\text{S}}} \right)$$

It is clear that the weight matrix in (4) is **K**, the bias is **F**^*H*^**F**, and the non-linear activation function is the shrinkage operator. The iterates in (5) produce a level-set estimate for a sufficiently deep network. The depth is necessary since the algorithm requires many iterations to converge. In practical applications, due to phase errors, the forward propagation produces defocused and smeared images. To suppress artifacts due to phase errors, we refine the ideal forward model **F** by learning **F**^*H*^ and **K** through the DAS algorithm.

While the current network architecture imitates image formation methods, it has limitations when it comes to supervised learning. In inverse imaging problems, supervised learning may not be applicable since the exact object generating the data, including precise tumor margins, is not fully known in real-world scenarios. Only the measured data and knowledge of the mapping are available. Additionally, the availability of training data may be limited. To address these challenges, we adopt an unsupervised approach using an autoencoder structure to learn from the data.

**Figure 2:**
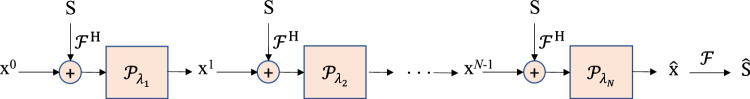
The recurrent auto-encoder architecture capable of training with measurements. The linear stages are represented as arrows. The non-linear activation functions are represented as boxes. At each layer, the linear gradient descent step is followed by the shrinkage operation.

To transform the above RNN architecture into an autoencoder, we add a new linear layer after the last RNN layer. This additional layer maps the estimated image back to the data using function **F**, enabling unsupervised training. The resulting architecture is a recurrent auto-encoder with *N* layers. The first (*N *− 1) layers are based on Eq. (5), while the final Nth layer performs forward propagation. Figure 2 illustrates the final architecture, where the regularization parameter can vary at each layer.

Although our training framework provides an effective approach, there are two challenges to consider. Firstly, storing the network parameters can be challenging due to the size of the images. Secondly, RNN training encounters the vanishing or exploding gradients problem, which arises when the weight matrix is poorly conditioned^60^. This issue is characterized by either oscillatory behavior or extremely slow convergence. To overcome this problem, the introduction of long short-term memory (LSTM) RNNs has proven beneficial^61^. LSTM consists of a memory cell along with input, output, and forget gates, which help address the exploding and vanishing gradient problem by modifying the image reconstruction process to be additive instead of multiplicative. LSTM is a popular RNN architecture known for its ability to store values from past instances and mitigate the issue of gradient diminishing. In the context of our imaging approach using wideband pulses, RNNs, including LSTM, are natural choices for processing time-series information.

## Experimental results

The effectiveness of our algorithm is first demonstrated by segmenting the reconstructed images of numerical phantoms containing tumors. The data are derived from our prior work on the development of skin-equivalent phantoms for mimicking interactions of millimeter waves with the human skin and skin tumors^62^. These phantoms closely mimic the dielectric properties of normal skin and cancer tissues at millimeter-wave frequencies. We test our algorithm to reconstruct the normal skin and the tumor tissue, while keeping the corresponding electrical properties similar to Ref.^62^.

**Figure 3:**
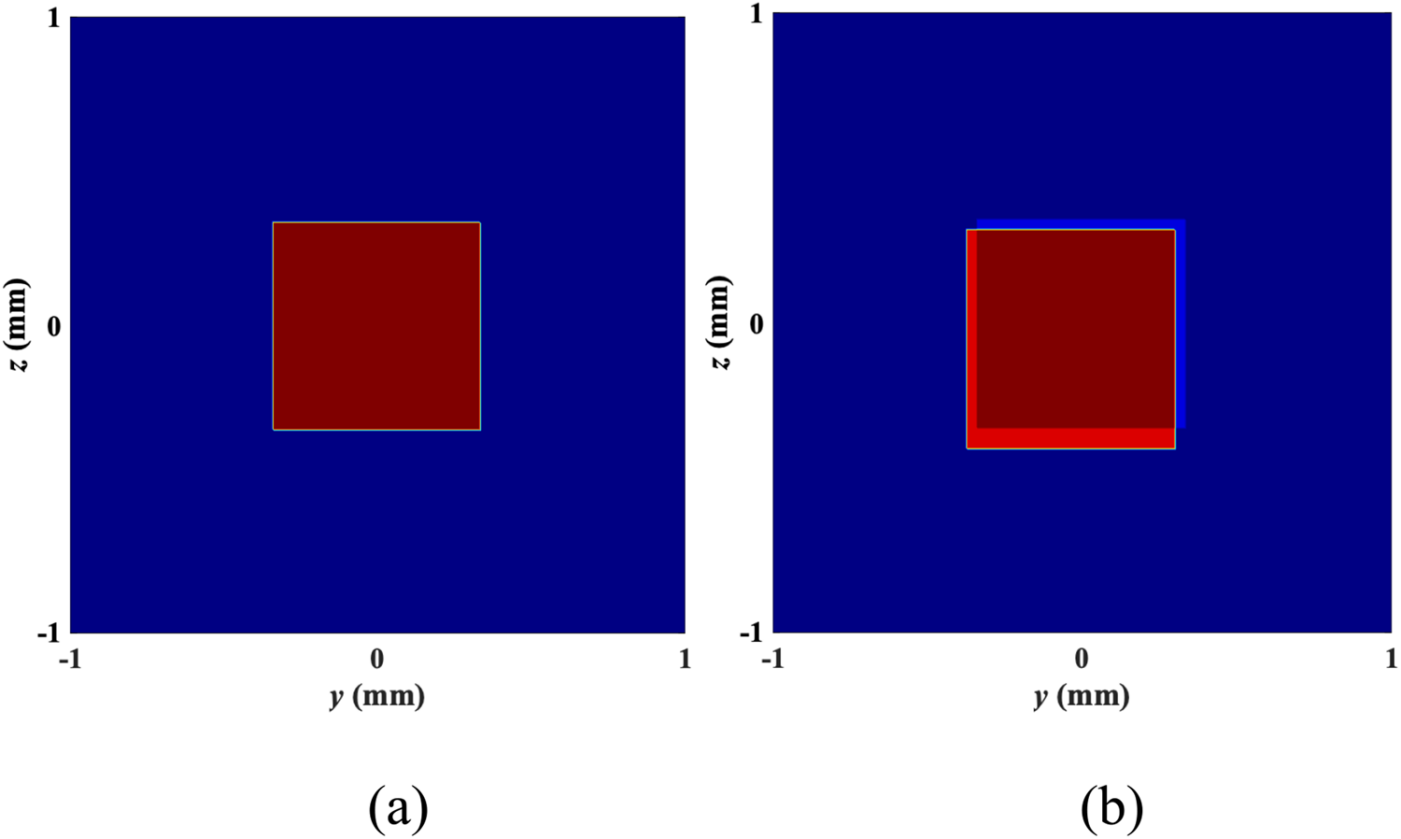
(**a**) Actual geometry. (**b**) Joint contrast and shape recovery.

We use a 2 × 2 m^2^ scene with a single stationary point target discretized into 30 × 30 pixels with the origin of the coordinate system located at the center of the scene as shown in Fig. 3a. The forward model explained in “Connection between DNN and nonlinear EM inverse scattering” was used to create a bistatic received signal. The circular trajectory was navigated by two antennas. The transmitted waveform had a bandwidth of 40 GHz and a center frequency of 90 GHz. To build a training set of 100 samples, we uniformly sampled the signal in slow time. Each measurement in the training set included additive phase error with a uniform distribution and a variance of 3 m, representing the antenna trajectory error. Additionally, a test set of 30 measurements was generated in the same manner. For our analysis, we constructed an 8-layer network following the architecture detailed in “DNN for nonlinear EM inverse scattering”.

**Figure 4:**
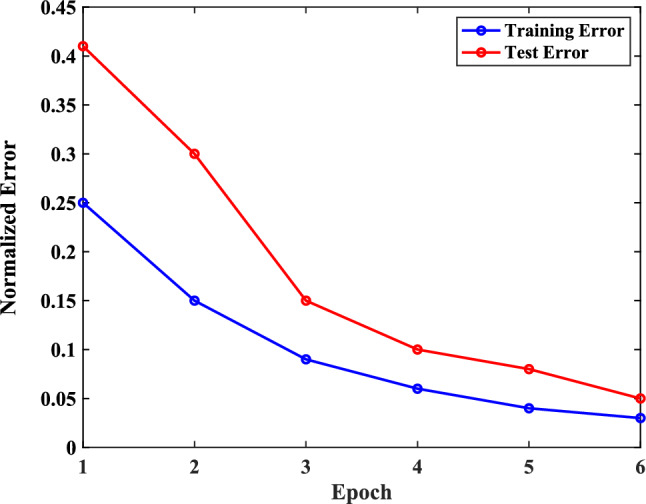
Averaged training and test loss vs epochs.

In our simulations, the network was trained for 6 epochs at which point we observed that the algorithm converged. We used the normalized $\mathcal{l}$^2^-norm error of the data residual as our metric. Figure 4 demonstrates the average test and training errors using 100 training samples and 30 test samples. As expected, the average errors for both groups decrease with respect to the epoch number. The test error decreases at a lower rate compared to the training error which is also typical.

For visual comparison between the results obtained, we display a reconstructed test image formed after the application of the proposed DeepTMI method. The reconstructed image is shown in Fig. 3b. To quantify our observations, we use the Intersection over Union (IoU) performance metric for shape estimation and the relative error metric to characterize the reflectivity function estimation. IoU is defined as the ratio of the volume of overlap between recovery and original to the volume of the union of the two. In the perfect shape recovery, IoU is 1. We use the average root-mean-square (rms) error as the second metric. The quantitative shape error for the cube is 5% and the relative error is 0.01%.

**Figure 5:**
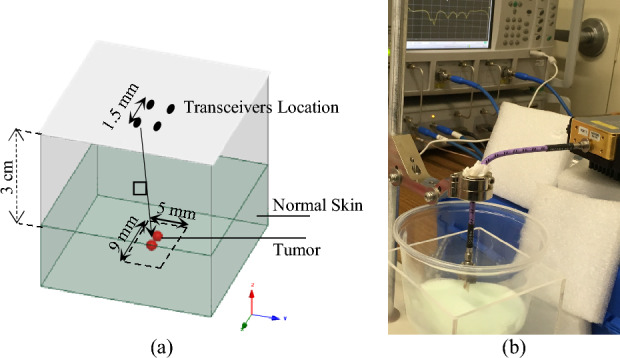
(**a**) Schematic layout and (**b**) testbed of experimental setup for evaluation of the performance of the proposed method.

The effectiveness of our algorithm is further demonstrated by segmenting the reconstructed images of numerical phantoms containing tumors. Experimental setups similar to Fig. 5a are considered for the evaluation of the performance of the proposed method. Data collection is performed in a monostatic manner in which the antennas are scanned over an aperture plane placed in front of the target. The operating frequency range of 75–110 GHz is considered. The antennas are SIW-based Vivaldi antennas previously developed by our group^63^. The antennas scan over the aperture plane across 2 × 2 positions in a rectangular pattern as shown in Fig. 5a. The spacing between consecutive scan positions is 1.5 mm, which is the maximum spacing that satisfies the Nyquist criterion. The distance between the aperture plane and the skin surface is 3 cm. The Nyquist criterion requires the maximum spacing of (~ λ/2) to be met between two consecutive scans. Considering the smallest wavelength of λ = 3 mm in our imaging system, the Nyquist spacing corresponds to 1.5 mm.

The phantoms were fabricated from our prior work on developing skin phantoms to mimic the interactions of electromagnetic waves with human skin and skin tumors^62^. These phantoms closely emulate the dielectric properties of normal skin and cancer tissues at millimeter-wave frequencies. Figure 5b shows the artificial phantoms fabricated and installed on the developed imaging testbed. Realistic skin phantoms serve as an invaluable tool for exploring the feasibility of new technologies and improving design concepts related to millimeter-wave skin cancer detection methods. Normal and malignant skin tissues are separately mimicked by using appropriate mixtures of deionized water, oil, gelatin powder, formaldehyde, TX-150 (a gelling agent, widely referred to as ‘super stuff’), and detergent.

Dispersive skin-mimicking phantoms available in the literature^62^ with a finite thickness of 2 mm were fabricated using a mixture of materials (water, gelatin, oil, salt, and surfactant) following the procedure presented in Ref.^62^. To emulate the resolution experiments, two canonical-spherical tumors with diameters of 400 µm were also fabricated and inserted within the phantoms. The tumor-mimicking mixture had 30% (in weight) higher oil content than the skin-mimicking mixture. This increase in oil percentage results in the closest possible dielectric properties to those of malignant skin tissues (specifically malignant SCC tissues) as reported in Ref.^64^.

We used single-pole Cole–Cole fitting to efficiently represent the measured data of biological tissues as:

6 $$\varepsilon_{r} (\omega ) = \varepsilon^{\prime}_{r} (\omega ) - j\varepsilon^{\prime\prime}_{r} (\omega ) = \varepsilon_{ro} (\omega ) + \frac{{\Delta \varepsilon_{r} }}{{1 + (j\omega \tau )^{1 - \alpha } }} + \frac{{\sigma_{s} }}{{j\omega \varepsilon_{0} }}$$

where* ε*_*r*_′(*ω*) and *ε*_r_″(*ω*) are the real and imaginary parts of the frequency-dependent relative dielectric permittivity (*ε*_*r*_(*ω*)), *ω* = 2π*f* (*f* is the frequency of operation), *j* = (− 1)^1/2^, *τ* is the relaxation time, Δ*ε*_*r*_ is the magnitude of the dielectric dispersion of the skin, *ε*_*r*o_ is the permittivity of skin at optical frequencies, *ε*_0_ is the permittivity of free space, *α* is a measure of the broadening of the dispersion, and *σ*_s_ is the ionic conductivity of the skin. The Cole–Cole parameters of the dielectric properties for each phantom are presented in Table 1.

**Table 1:** Cole–Cole parameters of the dielectric properties of the fabricated phantoms.

Parameter	*ε*_O_	Δ*ε*_*r*_	*τ *(*ps*)	*σ*_s_	*α*
Normal skin	4.25	45.02	5.96	0.0005	0.15
Malignant skin	10	34.99	3.04	0.0004	0.05

For the dataset, 50 arrangements were used for training. Different spherical-shaped tumor models with different sizes (diameters of 0.1–0.4 mm) and different distances (center distances 0.5–1 mm) are considered. The proposed network was trained using the Adam Optimization^65^. The regularization parameter was set to *λ* = 10^–7^ and the learning rate was set to 10^–4^. The number of hidden layers used was 8. In our experiments, the network was trained for 6 epochs at which point we observed that the algorithm converged.

**Figure 6:**
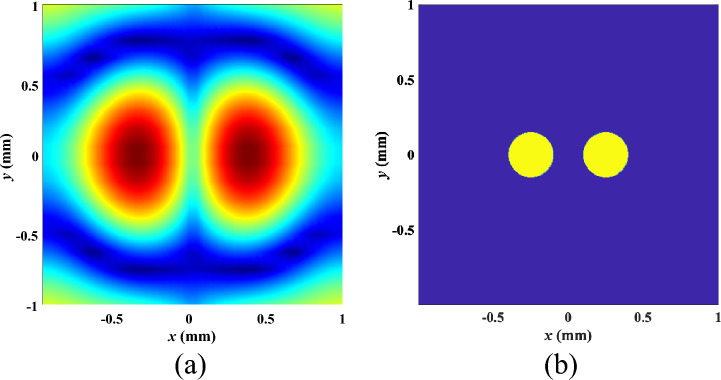
Image reconstructed by (**a**) the algorithm in Ref.^16^ and (**b**) the proposed deep learning-based level-set method.

For visual evaluation of the results obtained, in Fig. 6, we demonstrate the reconstructed image formed after the application of a reconstruction algorithm we developed in Ref.^16^ and the corresponding level-set function after application of the proposed learning method. The target consists of two spherical tumors as similar to Fig. 5. The defocusing effect when applying the conventional reconstruction algorithm is clearly displayed, which is also quantified by a poor T/C measure (the ratio of the peak tumor signal to the peak clutter). For this test sample, the T/C ratios are 5.5 dB and 22 dB for the images reconstructed using our conventional and the level-set approaches, respectively.

To quantify our observations, we use the structure similarity measure (SSIM) and mean-square-error (MSE) of the reconstructed images. SSIM and MSE are approximately 0.94 and 0.04 for the proposed method, while the values obtained from conventional frequency-domain reconstruction methods can hardly overcome 0.35 and 0.41, respectively.

## Discussion

In our previous imaging studies, we used an ultra-wideband image reconstruction technique developed in Ref.^16^ to create three-dimensional reconstructions of objects. Although the developed reconstruction technique takes the dispersive behavior of the target over the ultra-wide imaging bandwidth of nearly 100 GHz into account, it involves large amounts of data due to the high sampling rate needed to produce high-resolution images. This leads to an increased number of slow-time pulses and hence, increased computational complexity in the imaging algorithm. In addition to requiring high sampling rates, the algorithm obtains high-resolution images by transmitting pulses with ultra-wide bandwidths; the wider the pulse bandwidth in frequency, the narrower the pulse in time, improving depth resolution. Thus, it is desired to reduce the number of slow-time pulses and increase the bandwidth to maintain high-resolution imaging. However, the data acquisition process is frequently plagued by phase errors. Due to inaccuracies in the scanning measurement as well as possible existence of moving targets (such as arm and hand movements of patients during our clinical examinations) in the observed scene, the acquired data will contain phase errors. These phase errors in turn result in a defocusing effect in the formed images.

Many different algorithms have been developed over time to form images from backscattered data. These algorithms use several different approaches such as back projection^66^, compressed sensing^67^, or signal processing^68^. For sparse target scenes, adding the target detection process in the raw scattering process is an effective method to improve the imaging process. It is well known that the scattering data of a target at high frequencies can be represented as a sum of responses from individual scattering centers. These scatterers are sparsely distributed in the sensing scene i.e., most of the coefficients in the target’s signature vector are zeros or nearly zeros.

The theory of compressed sensing (CS) states that a high-dimensional signal can be accurately and robustly recovered from its lower-dimensional projections if the signal is sparse or can be sparsely represented. CS theory can be conveniently applied to many ultra-wideband (UWB) imaging applications due to the natural presence of sparsity in these problems^69^. Sparsity-based signal processing methods have achieved great success in both suppressing interference^70,71^ and performing spectral gap extrapolation^72,73^ to combat frequency notches. However, the available sparsity-based techniques in UWB imaging and sensing cannot accurately represent the diffraction scattering behavior for wide relative bandwidths since the model order cannot be estimated accurately in those situations.

To overcome the above-mentioned challenges, the method proposed in this paper offers an alternative approach to the traditional image formation algorithms. We solve the image formation problem in the presence of phase uncertainties. Such uncertainties arise due to target movement and errors in chip scanning trajectories among other sources. Using an iterative imaging algorithm, we will form a new neural network that emulates this process and further refines the parameters via training for improved performance.

In our proposed learning-based approach, as shown in Fig. 2, all steps of the image reconstruction processing are integrated into a deep neural network. Input to the network is raw scattering data, while the network output is the focused image. With our approach, all computational operations within the scheme are performed through the forward propagation of a single neural network. There is no need to pass processed data from one processing component to another, greatly simplifying the processing effort.

The main limitation of the presented work was the small size of the training dataset. In our future work, we will use more diverse experimental setups to train and evaluate the performance of the proposed method.

## Data Availability

The datasets used and/or analyzed during the current study available from the corresponding author on reasonable request.
